# Complete haplotype phasing of the MHC and KIR loci with targeted HaploSeq

**DOI:** 10.1186/s12864-015-1949-7

**Published:** 2015-11-05

**Authors:** Siddarth Selvaraj, Anthony D. Schmitt, Jesse R. Dixon, Bing Ren

**Affiliations:** Ludwig Institute for Cancer Research, La Jolla, CA 92093 USA; Biomedical Sciences Graduate Program, University of California San Diego, La Jolla, CA 92093 USA; Medical Scientist Training Program, University of California San Diego, La Jolla, CA 92093 USA; Department of Cellular and Molecular Medicine, and UCSD Moores Cancer Center, University of California San Diego, La Jolla, CA 92093 USA; Institute of Genomic Medicine, University of California San Diego, La Jolla, CA 92093 USA

**Keywords:** HaploSeq, MHC, HLA-Typing, KIR, Phasing

## Abstract

**Background:**

The MHC and KIR loci are clinically relevant regions of the genome. Typing the sequence of these loci has a wide range of applications including organ transplantation, drug discovery, pharmacogenomics and furthering fundamental research in immune genetics. Rapid advances in biochemical and next-generation sequencing (NGS) technologies have enabled several strategies for precise genotyping and phasing of candidate HLA alleles. Nonetheless, as typing of candidate HLA alleles alone reveals limited aspects of the genetics of MHC region, it is insufficient for the comprehensive utility of the aforementioned applications. For this reason, we believe phasing the entire MHC and KIR locus onto a single locus-spanning haplotype can be a critical improvement for better understanding transplantation biology.

**Results:**

Generating long-range (>1 Mb) phase information is traditionally very challenging. As proximity-ligation based methods of DNA sequencing preserves chromosome-span phase information, we have utilized this principle to demonstrate its utility towards generating full-length phasing of MHC and KIR loci in human samples. We accurately (~99 %) reconstruct the complete haplotypes for over 90 % of sequence variants (coding and non-coding) within these two loci that collectively span 4-megabases.

**Conclusions:**

By haplotyping a majority of coding and non-coding alleles at the MHC and KIR loci in a single assay, this method has the potential to assist transplantation matching and facilitate investigation of the genetic basis of human immunity and disease.

**Electronic supplementary material:**

The online version of this article (doi:10.1186/s12864-015-1949-7) contains supplementary material, which is available to authorized users.

## Background

The major histocompatibility complex (MHC) and the killer cell immunoglobulin-like receptor (KIR) are important regulators of human immune responses and are involved in many human diseases [1, 2]. These loci are highly polymorphic, allowing an extensive antigen-presenting repertoire that enables strong immunity against a wide range of foreign antigens, pathogens and tumor cells [1–3]. At the same time, its immunogenic heterogeneity can also create incompatibility in allotransplantation procedures, causing graft rejections and graft-versus-host disease (GVHD) [4, 5]. Furthermore, many of the hundreds of genes within these immunogenic loci are increasingly recognized as major susceptibility genes for drug hypersensitivity reactions and appear to play a significant role in numerous diseases, including cancer [6–8]. Taken together, the clinical implications of these loci make it useful to determine the sequence type of these molecules.

Typing of human leukocyte antigen (HLA) genes, located within the MHC locus, has traditionally been achieved in low resolution using serotyping techniques [9]. With advancements in technologies including PCR and more recently, next generation DNA sequencing (NGS), molecular-based methods have now enabled more clinically significant high-resolution HLA typing [10–12]. Notably, single-molecule NGS-based DNA sequencing has been demonstrated to resolve allele ambiguity by generating haplotypes of entire genes, resulting in super high-resolution (8-digit) haplotyping of HLA genes [13, 14]. However, even precise gene-level haplotyping may not be sufficient for many applications. For example, while gene-level haplotyping for several candidate HLA genes can reduce risk of graft failure in transplantation matching, recipients could still be susceptible to graft-versus-host disease, as the totality of transplantation associated genes have not been fully understood. In particular, reports suggest that non-HLA gene families such as inflammatory genes, immune receptors, or others across the MHC or KIR haplotype can contribute to transplantation biology [15–17]. In addition, the strong linkage disequilibrium (LD) patterns across the MHC and KIR loci can allow coordinated functional activities of alleles on the same haplotype, complicating our understanding of transplantation biology [4, 5, 9, 18, 19]. Indeed, knowledge of haplotypes across several HLA genes has been shown to generate improved transplantation outcome predictions [19, 20] and can therefore facilitate determination of novel haplotype patterns for drug discovery and genome-wide association studies [21]. In summary, it appears useful to haplotype the entirety of the MHC and KIR loci to enable better understanding of immune genetics through analyses of compound heterozygous alleles.

Several experimental protocols have been developed to construct long-range haplotypes. Specifically, methods have been developed to generate mega-base-sized haplotypes [22–25], while others can phase the entire chromosome [26–29]. However, the adaptability of these methods to generate user-defined targeted haplotypes is unclear. More recently, Targeted Locus Amplification (TLA) has been developed to accomplish targeted phasing [30], but as the haplotypes from TLA are limited to a few-hundred kilobases, they may not be amenable for phasing large mega-base scale loci such as the MHC. Here, we develop a method, referred to as targeted HaploSeq, to generate full-length complete haplotypes of MHC and KIR loci from a single assay. Specifically, targeted HaploSeq combines the previously published HaploSeq [26] method developed for genome-wide haplotype phasing, with oligo capture and sequencing. As a proof of principle, we have applied targeted HaploSeq to the MHC and KIR loci in human lymphoblastoid cells. We phased over 90 % of the alleles in MHC and KIR loci at an estimated accuracy of ~99 %. To our knowledge, targeted HaploSeq is the first method to phase the MHC and KIR loci into a single haplotype structure. These results establish the utility of targeted HaploSeq for MHC and KIR typing in biomedical research as well as clinical settings.

## Results and discussion

### Experimental design

**Fig. 1:**
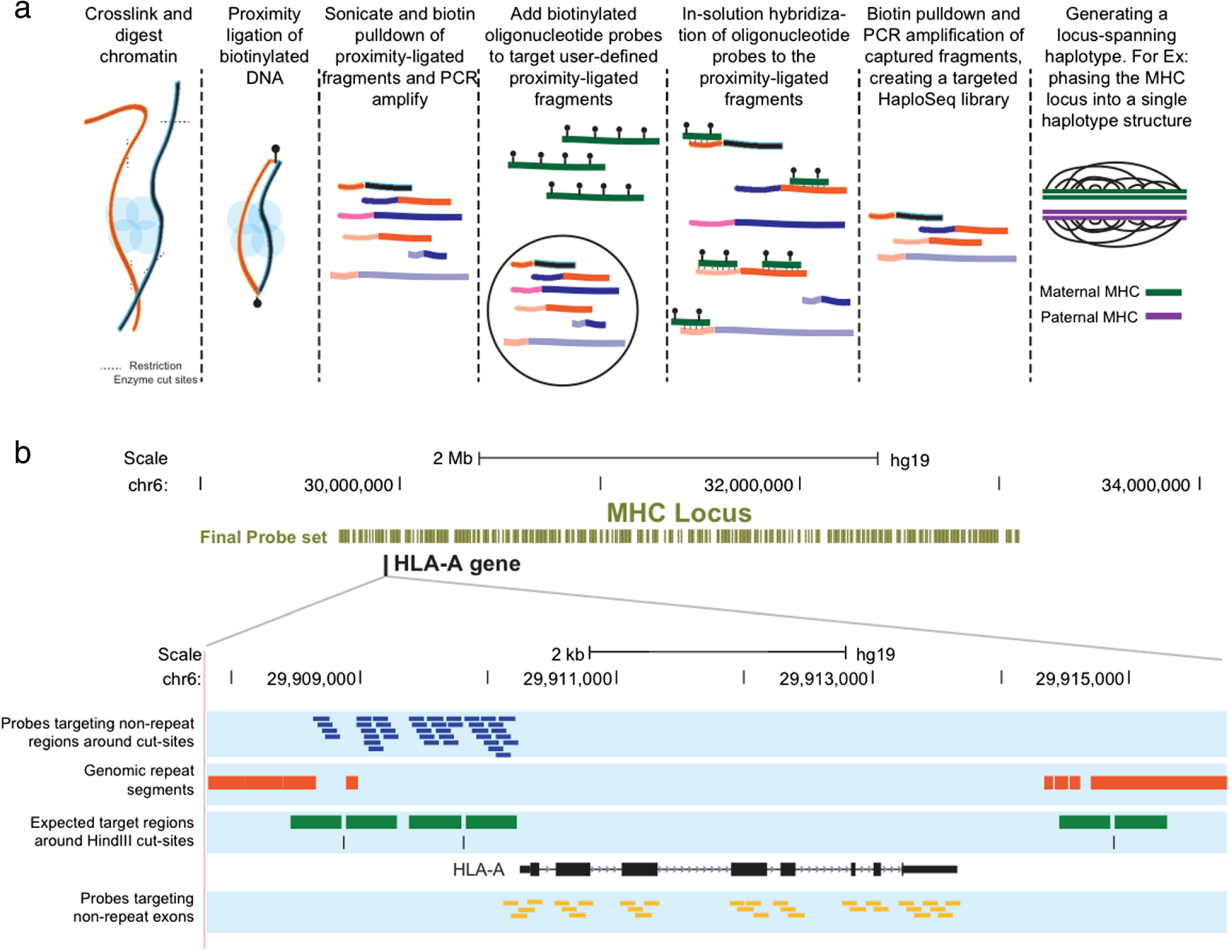
Targeted HaploSeq experimental design. **a** Outline of the Targeted HaploSeq protocol. Briefly, crosslinked chromatin is digested using restriction enzyme(s) of choice. The digested chromatin ends are biotinylated and ligated in a spatially proximal manner, enabling formation of signature artificial fragments—where spatially proximal distinct chromatin segments are combined into a single fragment. Target-specific oligonucleotide probes are then used to capture and enrich for user-defined proximity-ligated artificial fragments, to create a targeted HaploSeq library. This library is sequenced and used to generate locus-spanning haplotypes. **b** Illustration of oligonucleotide probe design: A browser shot of the 3.5 Mb MHC region illustrating location of probes near HindIII cut sites. The inset shows probe targets near HLA-A gene. Specifically, we tiled 120 nt probes (*blue*) at 4X density across non-repetitive segments around HindIII cut sites. In addition, we also targeted exonic regions of the MHC locus, as depicted in *yellow*

In the targeted HaploSeq method, a conventional Hi-C library [31] is generated using HindIII restriction digestion and amplified to obtain suitable material for oligonucleotide probe-based enrichment of the target loci (Fig. 1a). Briefly, based on simulation results (Additional file 1: Fig. S1), we computationally generated the probe sequences, at 4X tiling density, using the SureDesign Software (Agilent Technologies) and targeted the non-repetitive +/− 400 bp regions adjacent to HindIII cut sites over the MHC and KIR loci (Fig. 1b, Additional file 2: Fig. S2a). In addition, to facilitate better phasing of genic regions, we designed probes across the exons within the MHC locus (Fig. 1a).

**Fig. 2:**
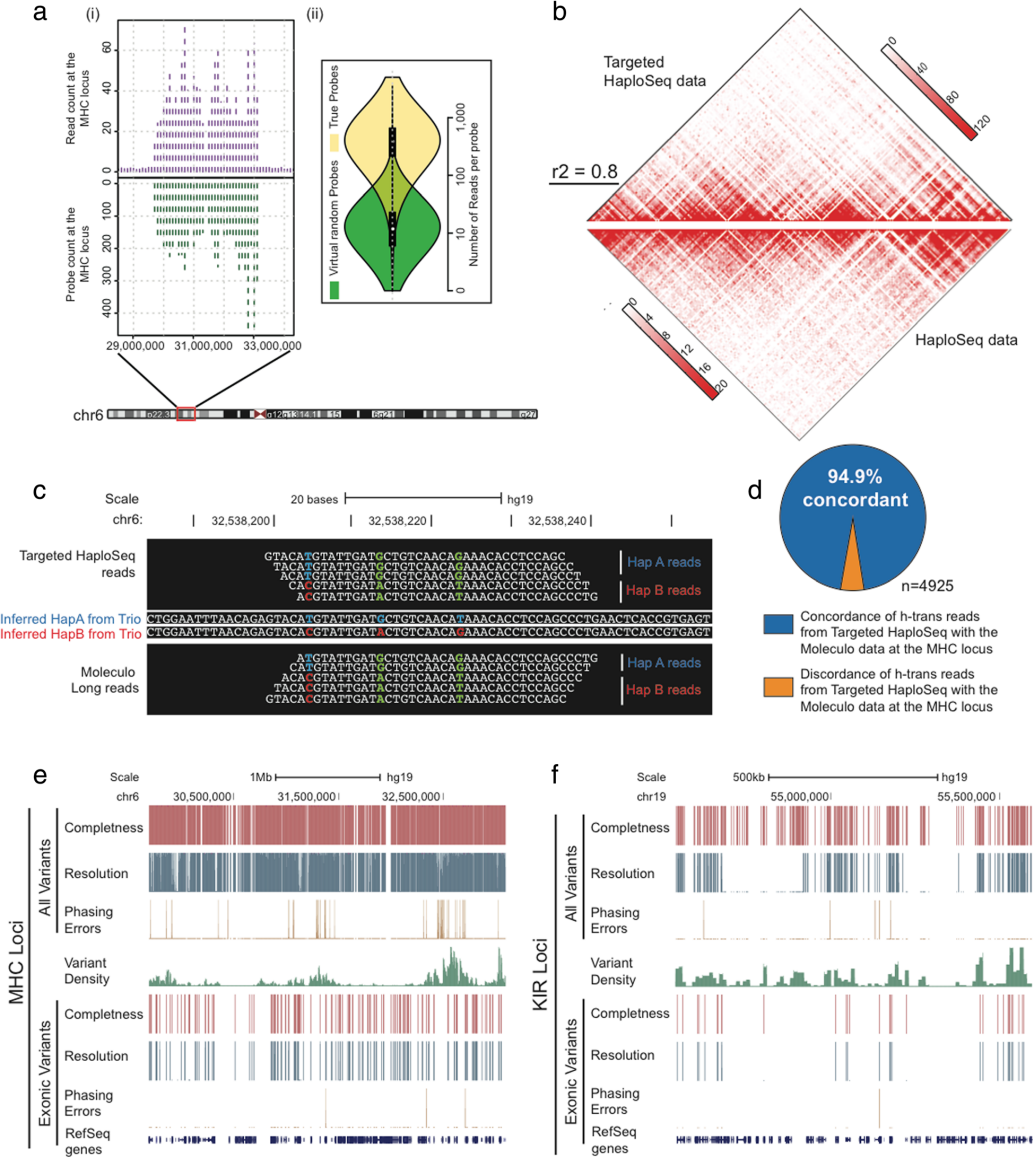
High-resolution and accurate phasing of MHC and KIR loci. **a** (i) Top chart demonstrates enrichment of targeted HaploSeq reads at the 100 kb binned MHC locus and the bottom plot shows number of probes in 100 kb bins used across the MHC locus. Visually, we can observe a high correlation between these plots, demonstrating the expected relationship between density of probes and the sequencing depth of targeted HaploSeq reads. (ii) To illustrate the sensitivity of probes, we virtually created random probes flanking HindIII cut sites and compared the enrichment in targeted HaploSeq data from these regions to the data from regions containing true probes. We observe ~100 fold more reads from true regions (on target, *yellow*) than the random regions (off target, *green*) and this fold-enrichment suggests high-sensitivity of our probes. **b** High correlation of targeted HaploSeq and the previously published HaploSeq datasets from GM12878 cells at the MHC locus (r^2^ = 0.8). **c** An example of haplotype inconsistency in the parent-child trio WGS data. Specifically, HapA (TGT-blue) and HapB (CAG-red) represent two haplotypes inferred from the trio dataset. Single-end reads from targeted HaploSeq (*top*) and Moleculo long-fragment reads (*bottom*) support a case of an inter-haplotype adjacent SNP-pair (*green*) and therefore raises an inconsistency with the parent-child trio haplotype inference. **d** Overall, ~95 % of the targeted HaploSeq reads representing homologous-trans (h-trans) interacting SNVs are concordant with the Moleculo LFR data. **e** High-resolution phasing capabilities of targeted HaploSeq method at the MHC locus. Completeness represents the collection of all heterozygous SNVs (*red*) within the MHC locus. Resolution represents the set of phased or resolved heterozygous SNVs in a single haplotype structure. While we observe ~1 % error, these errors are highly concentrated in the high variant density regions. The bottom section represents phasing of only exonic variants. **f** Similar figure as e) for the KIR locus

Next, by performing capture-sequencing [32, 33], we generated targeted HaploSeq data in GM12878 lymphoblastoid cells at 2× whole-genome sequencing depth with 30–50 fold target enrichment across the MHC and KIR loci (Fig. 2a, Additional file 2: Fig. S2b). More than 90 % of probes had at least 5-fold sequence coverage compared to data from virtual probes with an average of ~100 fold enrichment. This highlights the sensitivity of the probes from our targeted HaploSeq protocol. Next, to validate the quality of our targeted HaploSeq data, we compared it to a previously published HaploSeq dataset [26] generated from the same cell line. As HaploSeq utilizes chromatin interaction patterns to reconstruct haplotypes, we compared these between the two datasets and observed a high concordance (r^2^ = 0.8, Fig. 2b, Additional file 3: Fig. S3a, b). By using haplotype inference from the parent–child trio whole-genome sequencing (WGS) data [34], we examined the fraction of chromatin interactions between the homologous chromosomes (h-trans interactions), whose rarity is critical for accurate *de novo* haplotyping. Similar to HaploSeq, targeted HaploSeq data rarely exhibit h-trans interactions (Additional file 4: Fig. S4a).

Of note, the MHC locus appears to have a higher h-trans ratio in both HaploSeq and targeted HaploSeq datasets, but several lines of evidence suggest that these might be systematic errors from sequencing and analysis protocols. First, reads supporting h-trans interactions are primarily observed in complex regions with high variant density (Additional file 4: Fig. S4b). Second, >85 % of h-trans interactions from targeted HaploSeq dataset originate from the same end of a given paired-end fragment. Lastly, about 95 % of these same-end h-trans interactions are also observed in long-fragment reads (LFR) in previously published Moleculo datasets [25] from the same individual, indicating that a significant fraction of these h-trans interactions could have arisen from incorrect local haplotype inferences from the parent-child trio WGS data (Fig. 2c, d, Additional file 5). Taken together, our targeted HaploSeq data is of high quality and therefore enables accurate analyses of haplotype structures across the MHC and KIR loci.

### High-resolution and accurate phasing of MHC and KIR loci

By utilizing heterozygous genotype identifications (SNVs) from the trio-based WGS data [34], we used the HaploSeq and LCP protocols to perform *de novo* haplotyping. We generated a single haplotype structure over the MHC locus resolving over 90 % of ~9,400 heterozygous alleles and we used the trio-based haplotype structure to estimate the accuracy of our approach to be ~97.7 % (Additional file 6: Fig. S5). However, as the parent-child trio data could have accumulated incorrect phasing at regions with high variant density, we repeated the *de novo* haplotyping protocol after ignoring variants that we found to be h-trans in both our and LFR datasets. Consequently, our phasing accuracy improved to 98.94 % (Additional file 6: Fig. S5). Despite reducing the phasing error by over 50 %, from 2.3 to 1.06 %, we still observe a majority of phasing errors occurring in the high variant density regions (Fig. 2e). This suggests that the accuracy can potentially be further improved by using long-read or single molecule technologies that may be more suitable for mapping such complex regions. Of note, unlike switch errors—the standard method to calculate phasing error rates where an incorrect haplotype block is penalized only once, we estimate error by testing each variant independently and therefore our error rate represents worst-case scenario. To this end, as the density of variants affects the resolution of HaploSeq-based haplotyping, we observed a relatively lower resolution phasing for the KIR locus (Additional file 1: Fig. S1b). Regardless, we obtained accurate phasing of 348 out of 353 variants resolved at the KIR loci (Fig. 2f). Together, we resolved ~90 % of alleles among the MHC and KIR loci at ~99 % accuracy (Additional file 4: Fig. S4), demonstrating that our approach can generate complete, high-resolution and accurate haplotypes.

As current HLA typing protocols primarily type candidate genes across the MHC loci, we analyzed our method’s phasing capabilities across heterozygous genes from MHC and KIR loci. In total, we resolve ~92 % of heterozygous variants, representing over 92 % of heterozygous genes, at an accuracy of 99.34 % (Fig. 2e, f, Additional file 7: Fig. S6). In this regard, we generate highly accurate phasing for several “classical” genes used in conventional HLA typing protocols. For example, in the case of genes such as HLA-B, HLA-C, HLA-DRB1, HLA-DQA1, HLA-DQB1, HLA-DPA1 and HLA-DPB1, we resolve phasing of >99.5 % of the heterozygous variants at 100 % accuracy. Similarly at the KIR loci, we accurately predict all but one exonic variant (Additional file 7: Fig. S6). To our knowledge, our method is the first to demonstrate high-resolution and accurate haplotyping across the entire MHC and KIR loci, phasing not only the highly diverse major and minor alleles, but also other important immunological genes and variants at non-genic regions across the locus together in a single haplotype structure.

## Conclusions

Here, we describe the targeted HaploSeq method to generate large mega-base scale haplotypes in human cells. Using this technology, we reconstruct complete phase information of MHC and KIR loci. In principle, targeted HaploSeq is blind to genotyping and can be used to identify genetic variants *de novo* within the targeted loci. For example at the MHC locus, our method identified ~27 % of variants at an accuracy of 99.76 and 89.21 % for heterozygous and homozygous genotypes, respectively. This performance can be further improved with the use of multiple 4-base or 6-base cutters during Hi-C library preparation [35], instead of a single 6-base recognizing restriction enzyme as demonstrated in this manuscript. Alternatively, computational strategies such as population-based imputation can be also be used to generate comprehensive genotyping [36].

High-resolution genotyping and phasing of immunogenic loci such as MHC and KIR has several applications. First, it has the potential to greatly improve the practice of HLA typing/matching for clinical transplantation procedures [13, 15, 20, 37], as this method provides access to alleles that are otherwise un-typed using current methods. In addition, with population-scale MHC and KIR haplotyping, our method can help to elucidate a refined set of minimal alleles that confer the highest risk for GVHD, thereby informing follow-up cost-effective selective typing of these most informative alleles. Second, as our method phases coding and non-coding cis-regulatory sequences together, one can study patterns of compound heterozygosity and linkage of human immune variation [7, 16, 17]. Finally, several studies have uncovered numerous disease-associated HLA and KIR alleles and by understanding long-range haplotypes, we can now start to unravel mechanistic underpinnings of human immune disorders [21, 38, 39].

Recently, proximity-ligation methods such as Hi-C have been demonstrated to be useful in assembling genomes *de novo* [40, 41]. As targeted HaploSeq obtains high-quality chromatin interaction datasets, similar to Hi-C [31], this methodology can potentially be used to generate diploid assembly of complex regions, such as the MHC or T-cell receptor beta (Tcrb) locus [42], of human and other large genomes. Similarly, Hi-C has also recently been used in metagenomics studies to deconvolute the species present in complex microbiome mixtures [43, 44]. With the advent of targeted HaploSeq, it is now possible to capture distinct loci that are informative and discriminative enough to delineate species mixtures based on the captured proximity-ligation fragments.

Taken together, we present targeted HaploSeq and demonstrate its application for targeted phasing of HLA and KIR loci in the human genome. We believe that this method will lead to new avenues in biomedical research and in personalized clinical genomics.

## Data access

All sequencing data have been submitted to the Gene Expression Omnibus (GEO) database and will be publically available upon publication. Data has been made available under the accession number GSE65726.

## Ethics

Not applicable, non-human subjects.

## Additional files

Additional file 1: Figure S1. Targeting regions around HindIII cut sites allows complete and high-resolution haplotyping of MHC and KIR loci. a) (i) and (ii) depict completeness and resolution at MHC locus, respectively. We simulated reads across +/− 400 bp from HindIII cut sites in the MHC region to study our ability to obtain complete and high-resolution haplotypes. As the MHC region has a high-density of het. variants (a het. variance every ~300 bases), 2X sequencing coverage is enough to generate complete haplotypes, regardless of read length. On the same lines, we obtain high-resolution seed haplotypes at low sequencing coverage. b) (i) and (ii) depict completeness and resolution at KIR locus respectively. On the contrary, as the KIR locus has a lower density of variants, high sequencing coverage is required to obtain complete haplotypes. In particular, 40 bp reads are not enough to obtain complete phasing even at 50X coverage and therefore is omitted in the resolution plot. Similary, even at high sequencing coverage, resolution is very limited regardingless of read length. (TIFF 8219 kb) Additional file 2: Figure S2. Targeted enrichment at the KIR genomic locus. a) Genome browser shot of the ~1 Mb KIR region. The inset shows targets near KIR3DL2 gene, depicting target regions (green) around HindIII cut sites and repeat segments (red). We tiled 120-bp probes (blue) at 4X density accross these non-repeat target regions. b) (i) Top Plot demonstrates enrichment of GM12878 Targeted-HaploSeq reads at the 100 kb binned KIR locus while the bottom plot shows number of probes used across the KIR locus. Together, these plots show a high correlation among probes and read enrichment. (ii) Plot demonstrating sensitivity of capture probes—the true probes capture reads ~100 fold than random probes created virtually near HindIII cut sites (TIFF 8219 kb) Additional file 3: Figure S3. Targeted HaploSeq data has large pool of long insert fragments. a) Insert-size distribution of targeted Haploseq (green) and b) HaploSeq (purple) in GM12878 LCLs. Both these datasets have similar amount of long-insert fragments which is critical for long range haplotyping. (TIFF 8219 kb) Additional file 4: Figure S4. Homologous chromosomal interactions are rare and most of them are enriched in high variant density regions of the MHC loci. Using haplotypes indentified from the parent-trio whole genome sequencing data, we define homologous trans (h-trans) interactions in the Targeted Haploseq (green) and HaploSeq—from our previous publication (purple). a) h-trans interactions are rare −< 1 % in whole genome (i), about 5–6 % in the MHC locus (ii) and <0.5 % in KIR locus (iii). While h-trans interactions are <1 % whole-genome, we see them in significantly higher fractions at the MHC locus (~5 %). Interestingly, majority of these are found at regions with very high variant density (b), suggeting that the haplotype predictions from parent-trio data at these regions could be error-prone, which in-turn results in higher h-trans in HaploSeq datasets. (TIFF 8219 kb) Additional file 5:
**Online Methods.** (DOCX 149 kb) Additional file 6: Figure S6. Targeted HaploSeq generates a single (complete) haplotype structure across MHC/KIR locus. The performance metric of the Targeted HaploSeq protocol, measured by completeness (span of the haplotype bloc), resolution (fraction of het. alleles resolved), and accuracy. While each of these metrics were defined after performing read-based as well as population based haplotyping, seed resolution is estimated only based on read-based haplotyping. The overall resolution is defined as the weighted average among all alleles accross the MHC and KIR loci together. We observe over 50 % decrease in error rate from 2.3 to 1.06 % after correcting for potential incorrect local haplotypes from parent-trio data. (TIFF 8219 kb) Additional file 7: Figure S7. Targeted HaploSeq generates high quality phasing of heterozygous genes. Over 92 % of exonic het. variants are phased at an accuracy of 99 %. (TIFF 8219 kb)
