# Supplementary figures and images for: Complete haplotype phasing of the MHC and KIR loci with targeted HaploSeq
Source: BMC Genomics. 2015 Nov 5;16:900. doi: 10.1186/s12864-015-1949-7 (PMC4636068; doi:10.1186/s12864-015-1949-7)

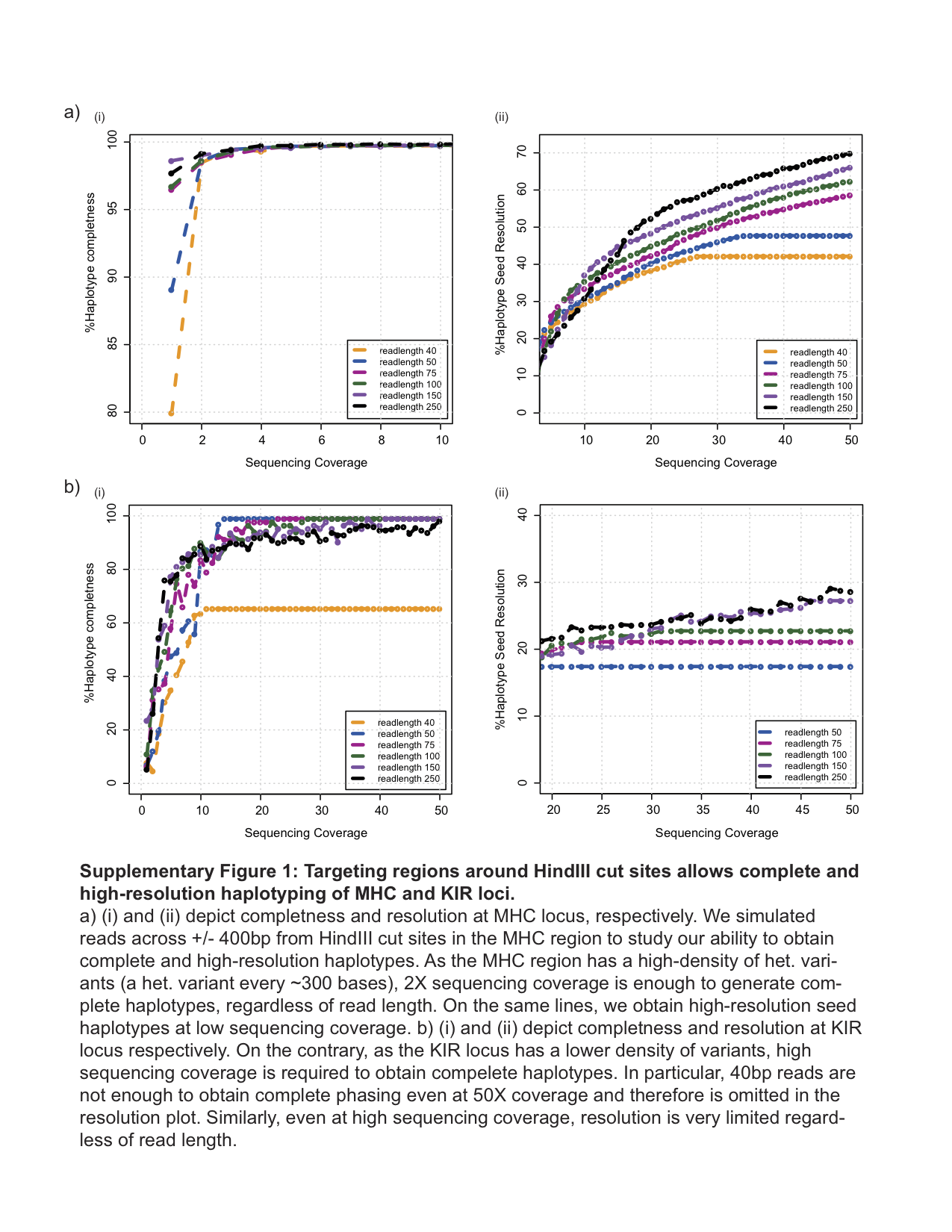

Supplement: Additional file 1: Figure S1. — Targeting regions around HindIII cut sites allows complete and high-resolution haplotyping of MHC and KIR loci. a) (i) and (ii) depict completeness and resolution at MHC locus, respectively. We simulated reads across +/− 400 bp from HindIII cut sites in the MHC region to study our ability to obtain complete and high-resolution haplotypes. As the MHC region has a high-density of het. variants (a het. variance every ~300 bases), 2X sequencing coverage is enough to generate complete haplotypes, regardless of read length. On the same lines, we obtain high-resolution seed haplotypes at low sequencing coverage. b) (i) and (ii) depict completeness and resolution at KIR locus respectively. On the contrary, as the KIR locus has a lower density of variants, high sequencing coverage is required to obtain complete haplotypes. In particular, 40 bp reads are not enough to obtain complete phasing even at 50X coverage and therefore is omitted in the resolution plot. Similary, even at high sequencing coverage, resolution is very limited regardingless of read length. (TIFF 8219 kb) [file 12864_2015_1949_MOESM1_ESM.tiff]

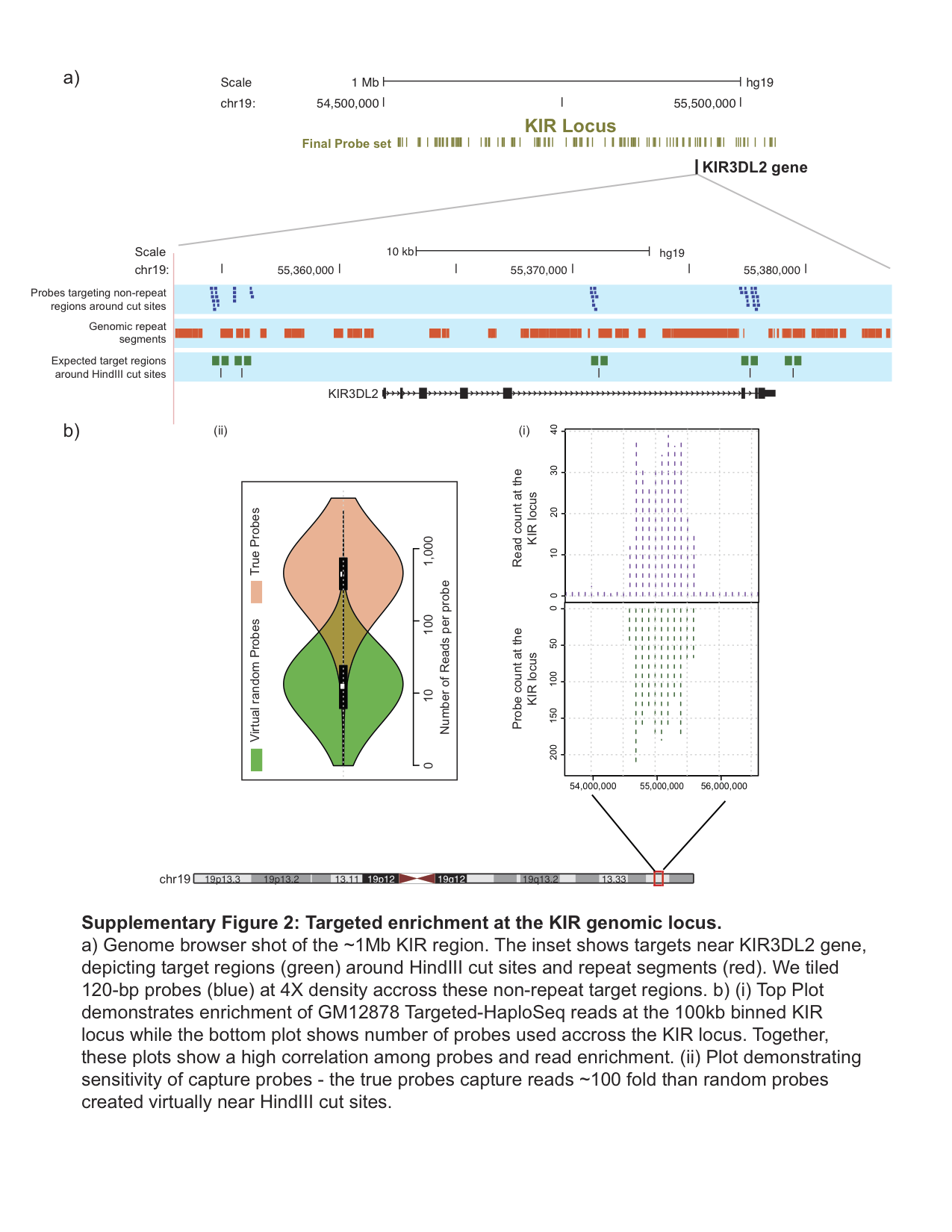

Supplement: Additional file 2: Figure S2. — Targeted enrichment at the KIR genomic locus. a) Genome browser shot of the ~1 Mb KIR region. The inset shows targets near KIR3DL2 gene, depicting target regions (green) around HindIII cut sites and repeat segments (red). We tiled 120-bp probes (blue) at 4X density accross these non-repeat target regions. b) (i) Top Plot demonstrates enrichment of GM12878 Targeted-HaploSeq reads at the 100 kb binned KIR locus while the bottom plot shows number of probes used across the KIR locus. Together, these plots show a high correlation among probes and read enrichment. (ii) Plot demonstrating sensitivity of capture probes—the true probes capture reads ~100 fold than random probes created virtually near HindIII cut sites (TIFF 8219 kb) [file 12864_2015_1949_MOESM2_ESM.tiff]

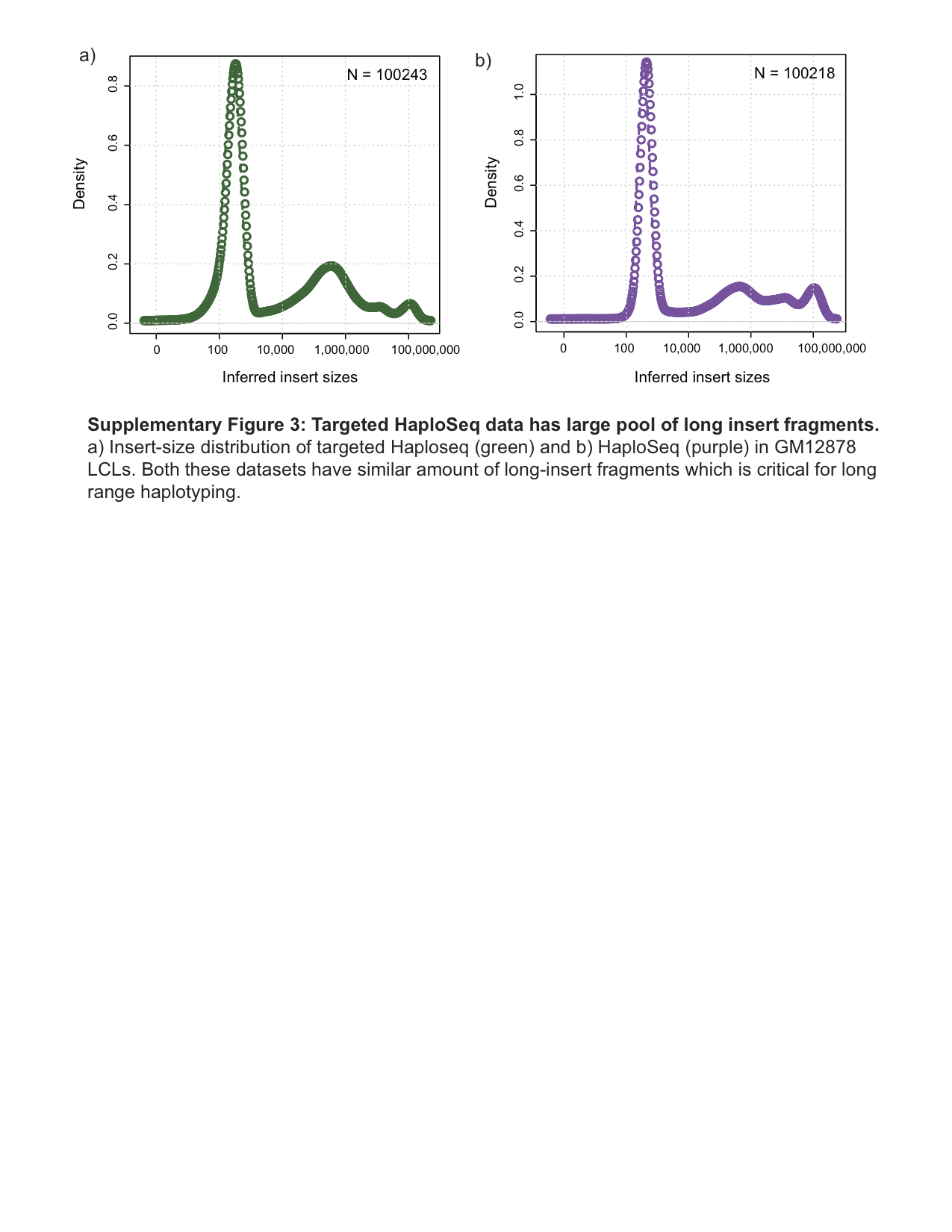

Supplement: Additional file 3: Figure S3. — Targeted HaploSeq data has large pool of long insert fragments. a) Insert-size distribution of targeted Haploseq (green) and b) HaploSeq (purple) in GM12878 LCLs. Both these datasets have similar amount of long-insert fragments which is critical for long range haplotyping. (TIFF 8219 kb) [file 12864_2015_1949_MOESM3_ESM.tiff]

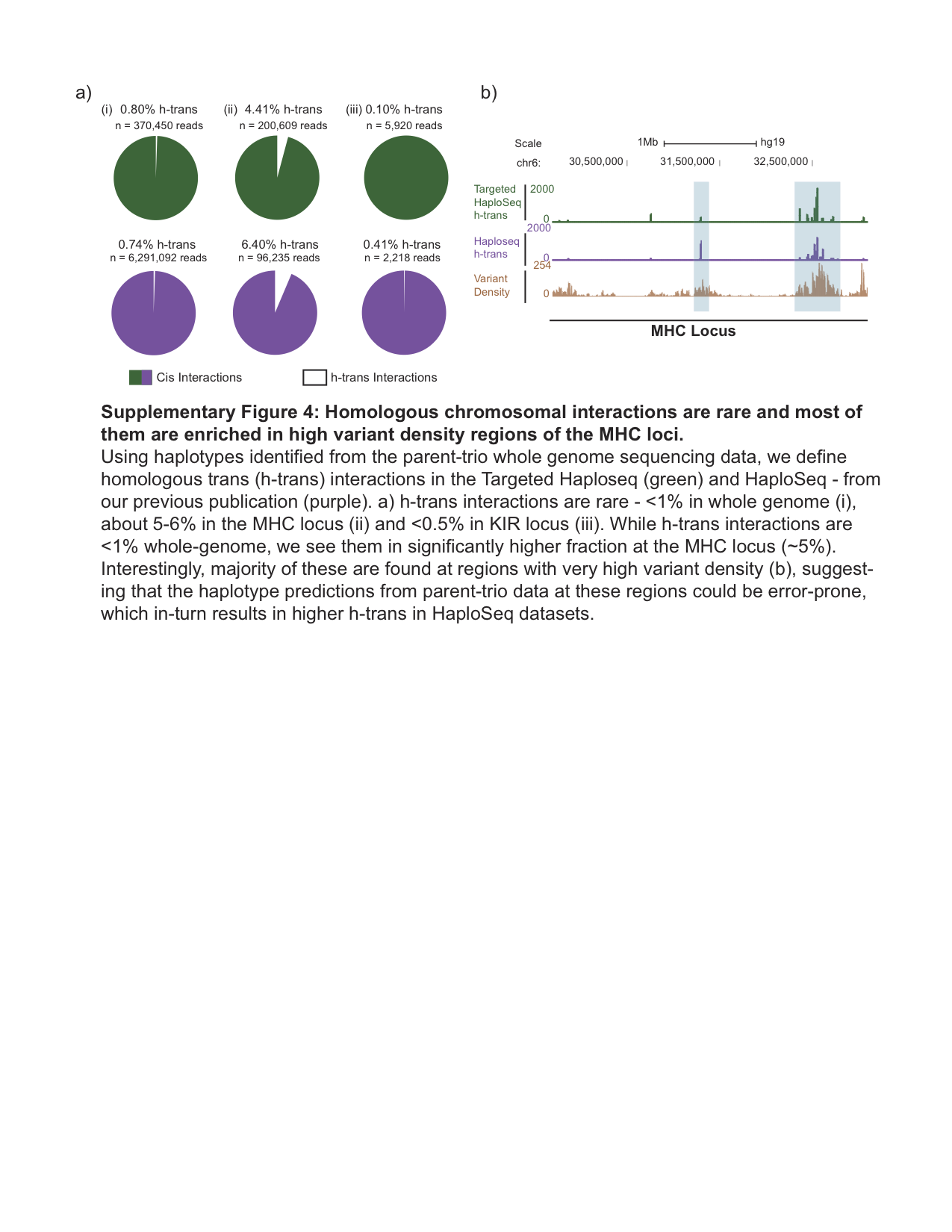

Supplement: Additional file 4: Figure S4. — Homologous chromosomal interactions are rare and most of them are enriched in high variant density regions of the MHC loci. Using haplotypes indentified from the parent-trio whole genome sequencing data, we define homologous trans (h-trans) interactions in the Targeted Haploseq (green) and HaploSeq—from our previous publication (purple). a) h-trans interactions are rare −< 1 % in whole genome (i), about 5–6 % in the MHC locus (ii) and <0.5 % in KIR locus (iii). While h-trans interactions are <1 % whole-genome, we see them in significantly higher fractions at the MHC locus (~5 %). Interestingly, majority of these are found at regions with very high variant density (b), suggeting that the haplotype predictions from parent-trio data at these regions could be error-prone, which in-turn results in higher h-trans in HaploSeq datasets. (TIFF 8219 kb) [file 12864_2015_1949_MOESM4_ESM.tiff]

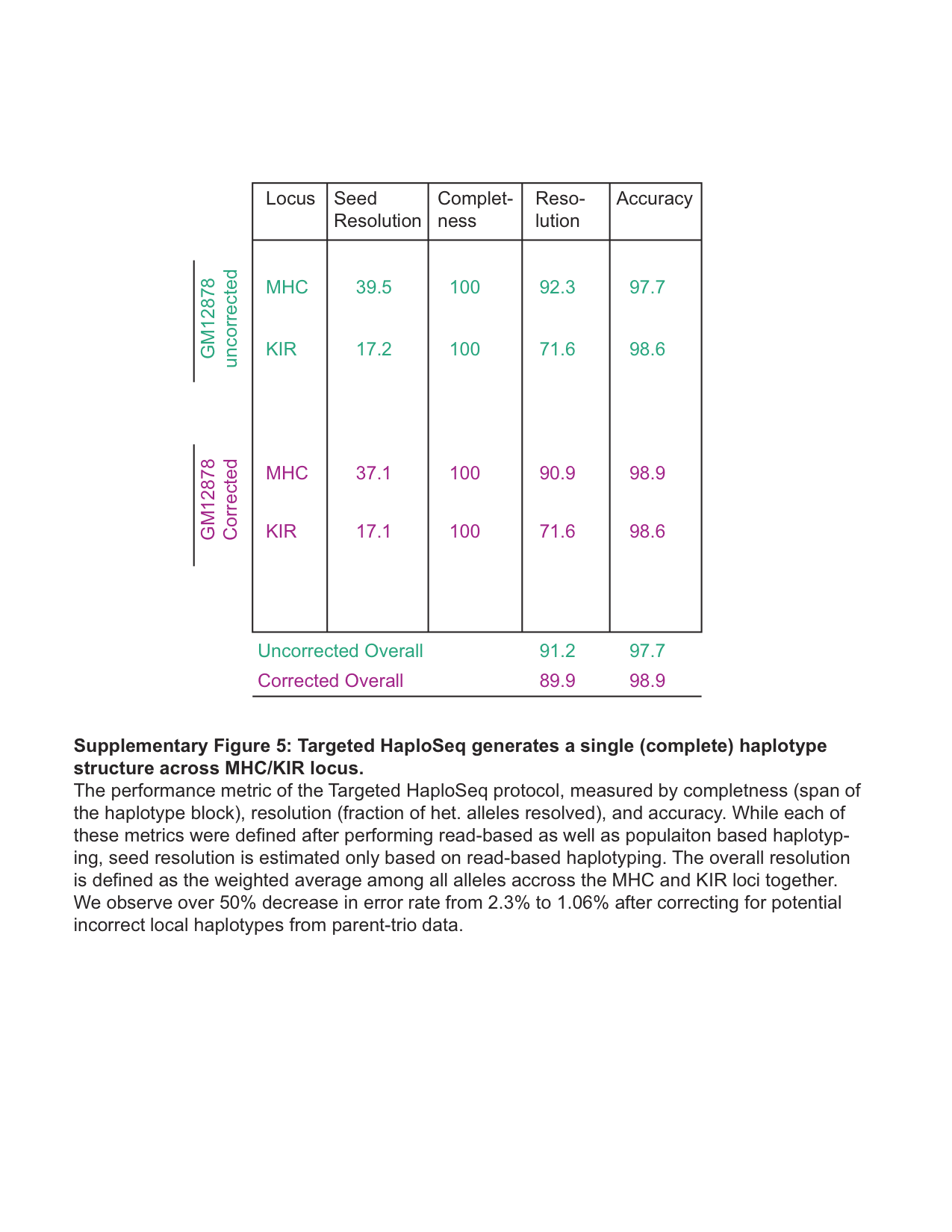

Supplement: Additional file 6: Figure S6. — Targeted HaploSeq generates a single (complete) haplotype structure across MHC/KIR locus. The performance metric of the Targeted HaploSeq protocol, measured by completeness (span of the haplotype bloc), resolution (fraction of het. alleles resolved), and accuracy. While each of these metrics were defined after performing read-based as well as population based haplotyping, seed resolution is estimated only based on read-based haplotyping. The overall resolution is defined as the weighted average among all alleles accross the MHC and KIR loci together. We observe over 50 % decrease in error rate from 2.3 to 1.06 % after correcting for potential incorrect local haplotypes from parent-trio data. (TIFF 8219 kb) [file 12864_2015_1949_MOESM6_ESM.tiff]

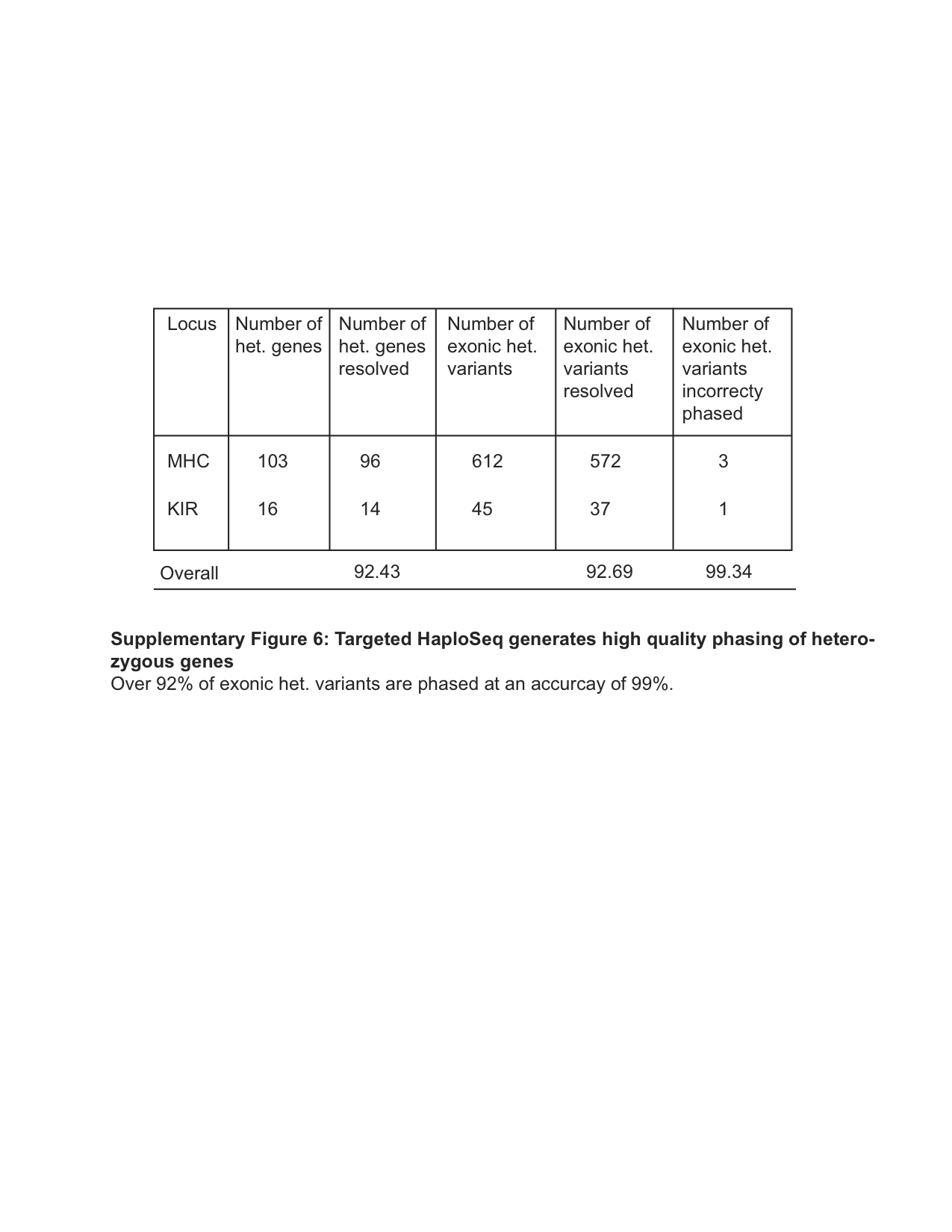

Supplement: Additional file 7: Figure S7. — Targeted HaploSeq generates high quality phasing of heterozygous genes. Over 92 % of exonic het. variants are phased at an accuracy of 99 %. (TIFF 8219 kb) [file 12864_2015_1949_MOESM7_ESM.tiff]
