# Supplementary material for: Complete haplotype phasing of the MHC and KIR loci with targeted HaploSeq
Source: BMC Genomics. 2015 Nov 5;16:900. doi: 10.1186/s12864-015-1949-7 (PMC4636068; doi:10.1186/s12864-015-1949-7)
Supplement: Additional file 5: — Online Methods. (DOCX 149 kb) [file 12864_2015_1949_MOESM5_ESM.docx]

**Generation of Hi-C libraries.** GM12878 (Coriell) cells were cultured in suspension in 85% RPMI media supplemented with 15% FBS and 1X penicillin/streptomycin. Cells were harvested, fixed in 1% formaldehyde, and 5 million cells were subject to the Hi-C protocol as previously described[^1^](#_ENREF_1). Prior to target enrichment, Hi-C libraries were amplified by 14 cycles of PCR using a high-fidelity (Fusion) polymerase. The number of pre-enrichment PCR cycles can be tailored depending on how much DNA is required for downstream target hybridization reactions. In this case, we performed several parallel PCR reactions using small amounts of bead-bound Hi-C library input at 14 cycles to obtain sufficient material.

**Generation of RNA baits.** Probes were computationally designed using the SureDesign software suite (Agilent Technologies). The custom design targeted the upstream and downstream 400bp adjacent to HindIII cut sites spanning the MHC (chr6:29689001-33098550) and KIR (chr19:54538900-55596120) loci using the hg19 genome build. SureDesign parameters were set to 4X tiling density, maximum probe boosting, and maximum repetitive sequence masking. We also targeted MHC exons at 2X tiling density, balanced boosting, and maximum repetitive element masking. In sum, 8,702 unique probes sequences were computationally generated, of which 6,413 correspond to regions flanking HindIII cut sites across the MHC locus, 765 correspond to HLA exons, and 1,524 probes were allocated to regions flanking HindIII cut sites across the KIR locus. In total, 12,298 probes were synthesized after considering some probes are duplicated in the design due to the boosting parameter of SureDesign. Using the probe sequences output by SureDesign, single-stranded DNA (ssDNA) oligos were synthesized by CustomArray Inc. ssDNA oligos contained universal forward and reverse priming sequences. Universal forward priming sequences contained a truncated SP6 RNA polymerase recognition sequence that is engineering to be completed upon PCR amplification of the ssDNA oligos. The reverse universal priming sequence contained a BsrDI recognition sequence for 3’ dsDNA probe cleavage prior to *in vitro* transcription. To convert ssDNA oligos into biotinylated RNA baits, ssDNA oligos were first diluted to 200pg/ul and then PCR-amplified using high-fidelity DNA polymerase (KAPA) and purified using Ampure XP beads (Beckman Coulter). As mentioned, the PCR reaction also serves the purpose to complete the remainder of the 5’ SP6 recognition sequence. Next, reverse priming sequences were removed by digesting the dsDNA with BsrDI (New England Biosciences) and purified again using Ampure XP beads to remove the digested fragments. Lastly, we performed *in vitro* transcription (IVT) according to manufacturers protocol (Ambion) in the presence of biotinylated UTP (Epicentre). RNA was then column-purified (Qiagen), diluted to working concentration (500ng/ul) and stored at -80 until use.

**Generation of targeted HaploSeq libraries.** To enrich our Hi-C libraries for proximity ligation fragments mapping to the MHC and KIR loci, we performed target enrichment using our custom RNA baits, followed by PCR amplification and sequencing. Briefly, 500ng of Hi-C library was incubated overnight at 65 degrees with 500ng of biotinylated RNA probe along with 2.5ug of human Cot-1 DNA (Life Technologies), 2.5ug Salmon Sperm DNA (Life Technologies) and blocking primers at a final concentration of 6.67uM. Next, RNA:DNA hybrids were isolated using T1 streptavidin-coated beads (Invitrogen) while DNA molecules not bound by RNA baits were washed away. Finally, captured products were resuspended in water and PCR amplified (Fusion) on-bead using 10-11 cycles. Lastly, PCR products were purified using AMPure XP beads (Beckman Coulter) and then subject to next-generation sequencing on Illumina HiSeq2500 to obtain approximately 51M reads pairs.

**Genotyping.** Variant calls and genotypes for GM12878 were downloaded from 1000 genomes project[^2^](#_ENREF_2) and these were used for *de novo* haplotype reconstruction from targeted HaploSeq data. Predicted haplotypes were compared with phasing Information for GM12878 from the 1000 genomes project to estimate accuracy.

**Read Alignment.** We mapped targeted HaploSeq reads to the hg19 genome. Reads were aligned using BWA Mem[^3^](#_ENREF_3) (Version: 0.7.5a-r405) as single end and these reads were manually paired using in-house scripts. Unmapped reads were removed and PCR duplicate reads were removed using Picard (Version: 1.49). The aligned datasets were then finally processed with GATK[^4^](#_ENREF_4) (Version: March 2013) for indel realignment and variant recalibration. The alignment process resulted in ~86% of reads mapping to genome in a non-duplicative fashion, of which about 7% of the reads mapped to the MHC/KIR loci.

**Simulations of haplotype completeness and resolution over MHC and KIR loci.** Here, we used different combinations of read length and coverage to obtain predictions at various haplotyping resolutions (Supplementary Figure 1). In particular, we used read lengths of 40, 50, 75, 100, 150 and 250 bases at coverage up to 50X of the MHC and KIR loci. To maintain the Hi-C insert distributions, we used human H1 Hi-C[^5^](#_ENREF_5) intra-chromosomal read starting positions at the MHC/KIR locus where at least one end of the pair-end is within 400bp from the HindIII cut-sites. We constructed graphs with nodes representing heterozygous variants in GM12878 (MHC/KIR) and edges corresponding to reads that cover multiple variants. These graphs allowed to us to predict completeness and resolution of the haplotypes through simulated data. Specifically, if the edge covers from “first” to the “last” SNP, the graph is 100% complete and the fraction of SNPs covered or phased in the longest graph is termed resolution. From various simulations (Supplementary Figure 1), the high density of heterozygous variants in the MHC loci allows complete and high-resolution haplotypes with low sequencing coverage. On the other hand, longer reads are necessary to obtain complete haplotypes in the KIR loci.

**Target Enrichment violin plots.** The violin plots (Figure 2aii and Supplementary Figure 2bii) represent reads per probe in two cases. In the first case, we take the actual probe locations in the MHC and KIR locus and estimate the read count per probe. In the second case, we designed “virtual” probes randomly across the genome (excluding the MHC and KIR loci) but confined to regions adjacent to HindIII cut sites similar to actual probes. We then counted reads per virtual probe. A 100-fold difference between read counts in these two cases demonstrates that our oligonucleotide based capture is sensitive in obtaining data from the targeted loci.

**Homologous Trans interaction estimations.** We used haplotype predictions from parent-child trio dataset[^2^](#_ENREF_2) to estimate the fraction of *cis* and fraction of homologous *trans* interaction (read1 and read2 map between the two homologous chromosomes) from the targeted HaploSeq data and the HaploSeq data. As reported earlier in the HaploSeq method[^6^](#_ENREF_6), we observe rare homologous interactions genome-wide (excluding MHC locus, Supplementary Figure 4ai). However, the fraction of homologous *trans* (or h-trans) is notably higher in the MHC locus (Supplementary Figure 4aii). Interestingly, the vast majority of these h-trans interactions from the MHC locus occur in regions with very high heterozygous variant density (Supplementary Figure 4b).

**Haplotyping.** Using variant calls and genotypes from the 1000 genomes project[^2^](#_ENREF_2), we used HaploSeq protocol[^6^](#_ENREF_6) to generate locus-spanning haplotypes across the MHC and KIR locus.

**Using Moleculo LFR data to decipher discordancy in haplotype calls.** We downloaded raw reads from Moleculo LFR data from GM12878 cells[^7^](#_ENREF_7). We adopted the Hi-C mapping strategy for Moleculo data. A snapshot of the data is described in Figure 2c. In order to estimate an overall rate for concordance among targeted HaploSeq and Moleculo data regarding the inter-haplotypes from parent-trio data, we did the following; Using the haplotype predictions from parent-child trio data, we first grouped all the SNP-pairs that represented inter-haplotype interactions from targeted HaploSeq. Then, for all these SNP-pairs, we asked what fraction of them is also inter-haplotype from Moleculo data. This fraction was approximately 67%. However, it turns out that these 67% of SNP-pairs are the well-supported inter-haplotype junctures, as they represent ~95% the inter-haplotype reads from targeted HaploSeq. In other words, 95% of inter-haplotype reads from the targeted HaploSeq data are concordant with molecule data (Figure 2d). Consequently, these SNPs from these SNP-pairs were removed from the genotype list and the haplotyping analysis was repeated, resulting in more than a 50% reduction in haplotyping error (Supplementary Figure 5).

**Exon Phasing.** We downloaded RefSeq gene list for hg19 build and kept only the longest transcript for a given gene in both the MHC and KIR locus. Therefore, each gene is represented by only one canonical transcript and the heterozygous variants from the exons of these canonical transcripts were used for the haplotyping analysis (Figure 2e,f and Supplementary Figure 6).

**Genotype Predictions using targeted HaploSeq data.** We used GATK pipeline and UnifiedGenotyper to call variants. We retained genotype predictions that had a sequencing depth of at least 10 and had a PASS filter after the GATK variant recalibration step.

**References**

1 Lieberman-Aiden, E. *et al.* Comprehensive mapping of long-range interactions reveals folding principles of the human genome. *Science* **326**, 289-293, doi:10.1126/science.1181369 (2009).

2 Genomes Project, C. *et al.* A map of human genome variation from population-scale sequencing. *Nature* **467**, 1061-1073, doi:10.1038/nature09534 (2010).

3 Li, H. Aligning sequence reads, clone sequences and assembly contigs with BWA-MEM. *Preprint at arXiV:1303.3997v2 [q-bio.GN]* (2013).

4 DePristo, M. A. *et al.* A framework for variation discovery and genotyping using next-generation DNA sequencing data. *Nature genetics* **43**, 491-498, doi:10.1038/ng.806 (2011).

5 Dixon, J. R. *et al.* Chromatin architecture reorganization during stem cell differentiation. *Nature* **518**, 331-336, doi:10.1038/nature14222 (2015).

6 Selvaraj, S., J, R. D., Bansal, V. & Ren, B. Whole-genome haplotype reconstruction using proximity-ligation and shotgun sequencing. *Nat Biotechnol* **31**, 1111-1118, doi:10.1038/nbt.2728 (2013).

7 Kuleshov, V. *et al.* Whole-genome haplotyping using long reads and statistical methods. *Nature biotechnology* **32**, 261-266, doi:10.1038/nbt.2833 (2014).
